# Understanding viral shedding of severe acute respiratory coronavirus virus 2 (SARS-CoV-2): Review of current literature

**DOI:** 10.1017/ice.2020.1273

**Published:** 2020-10-20

**Authors:** Lauren M. Fontana, Angela Holly Villamagna, Monica K. Sikka, Jessina C. McGregor

**Affiliations:** 1 Department of Medicine, University of Minnesota Infectious Diseases and International Medicine, Minneapolis, MN, USA; 2 Division of Infectious Diseases, Department of Medicine, School of Medicine, Oregon Health & Science University, Portland, Oregon; 3 Department of Pharmacy Practice, College of Pharmacy, Oregon State University, Portland, Oregon

## Abstract

**Objective:**

Transmission of SARS-CoV-2 has significant implications for hospital infection prevention and control, discharge management, and public health. We reviewed available literature to reach an evidenced-based consensus on the expected duration of viral shedding.

**Design:**

We queried 4 scholarly repositories and search engines for studies reporting SARS-CoV-2 viral shedding dynamics by PCR and/or culture available through September 8, 2020. We calculated the pooled median duration of viral RNA shedding from respiratory and fecal sources.

**Results:**

The review included 77 studies on SARS-CoV-2. All studies reported PCR-based testing and 12 also included viral culture data. Among 28 studies, the overall pooled median duration of RNA shedding from respiratory sources was 18.4 days (95% CI, 15.5–21.3; I^2^ = 98.87%; *P* < .01). When stratified by disease severity, the pooled median duration of viral RNA shedding from respiratory sources was 19.8 days (95% CI, 16.2–23.5; I^2^ = 96.42%; *P* < .01) among severely ill patients and 17.2 days (95% CI, 14.0–20.5; I^2^ = 95.64%; *P* < .01) in mild-to-moderate illness. Viral RNA was detected up to 92 days after symptom onset. Viable virus was isolated by culture from −6 to 20 days relative to symptom onset.

**Conclusions:**

SARS-COV-2 RNA shedding can be prolonged, yet high heterogeneity exists. Detection of viral RNA may not correlate with infectivity since available viral culture data suggests shorter durations of shedding of viable virus. Additional data are needed to determine the duration of shedding of viable virus and the implications for risk of transmission.

Knowledge of transmission dynamics of severe acute respiratory syndrome coronavirus 2 (SARS-CoV-2) has significant implications for hospital infection prevention and control interventions, timely discharge management, and public health policies. Due to variability in the emerging data, policies on the duration of inpatient and outpatient isolation for people with coronavirus disease 2019 (COVID-19) have been controversial. Uncertainty continues regarding the significance of prolonged PCR positivity and the clinical importance of various routes of viral shedding. Understanding the duration and sources of viable viral shedding is critical to inform guidance around transmission-based isolation precautions.

We reviewed SARS-CoV-2 viral shedding data to help inform practical decisions related to infection control and public health policies. We reviewed available literature and summarized data on expected duration of viral RNA shedding, longevity of presumed infectivity as detected by viral culture, and factors that may influence shedding duration.

## Methods

### Search method and data extraction

We queried PubMed, LitCoVID, the World Health Organization COVID-19 literature repository, and Google Scholar for studies and reports available through September 8, 2020. Search terms included of “SARS shedding,” “COVID and viral shedding,” “COVID RNA and culture,” and “COVID culture.” In queries of SARS-CoV-2–specific databases, the words “SARS” and “COVID” were omitted from search terms. Additional studies were identified through review of reference lists of included studies. All authors participated in study identification, screening, and data extraction; all included studies were reviewed by at least 2 authors. Articles reporting duration of SARS-CoV-2 shedding based upon PCR testing or culture directly from human specimens were included. Day 0 was defined as either the day of the first positive test or the day of symptom onset, according to the original study. Studies reporting on exclusively pediatric patients were excluded. For each study, we reviewed the design, objective, population, healthcare system setting, diagnostic testing method, timing of tests, sample source, patient symptoms, and severity of illness. Predictors of prolonged shedding were also considered.

### Statistical analysis

We constructed random-effects models using the restricted maximum likelihood estimator for τ ^2^ to calculate pooled median durations of viral RNA shedding.^1^ All studies providing sample size and sufficient data on measures of central tendency and spread were included in our analysis. We grouped nasopharyngeal (NP), oropharyngeal (OP), saliva, and sputum samples together as “respiratory” samples. Fecal samples included both stool and rectal swabs. We calculated pooled medians among PCR respiratory samples for all available, mild-to-moderate illness, severe-to-critical illness, and for all fecal samples. Insufficient data were available to warrant calculation of pooled medians for culture data. Analysis was performed using R version 4.0.0 software^2^ using the metamedian package.^3^

## Results

### Included studies

In total, 77 studies and reports were eligible for inclusion: prospective case series (N = 35), retrospective case series (N = 28), case reports (N = 11), point prevalence survey (N = 2), and position statements (N = 1) (Table 1). Overall, 59 of these studies were peer reviewed, 6 were from preprint servers, and 13 were research letters or letters to the editor. Moreover, 70 studies described hospitalized patients. All studies reported PCR-based assessments of viral shedding; 12 studies reviewed reported viral culture data.^4-15^ Also, 30 studies reported PCR testing of nonrespiratory specimens.

**Table 1:** Summary of Literature Included in Review of SARS-CoV-2 Viral Shedding

Study	Country	Design	Patient Population	No. of Patients	Severity of Illness	Specimen Sources	Testing Methods
Andersson M et al^6^	United Kingdom	Prospective case series	Hospitalized, outpatient, recovered	278	Not defined	Serum	PCR Viral culture
Arashiro T et al^60^	Japan	Case report	Hospitalized	2	Mild	Not specified	PCR
Arons MM et al^9^	United States	Point prevalence	Skilled nursing facility residents	76	Not defined	NP OP	PCR Viral culture if PCR +
Bullard J et al^5^	Canada	Retrospective cross-sectional	Not specified	90	Not defined	NP ET tube	PCR Viral culture
Campioli C et al^26^	United States	Retrospective case series	Hospitalized, outpatient	251	Not defined	NP	PCR
Chang M et al^46^	China	Prospective case series	Hospitalized	16	Not defined	Throat	PCR
Chau NVV et al^18^	Vietnam	Prospective case series	Quarantine center	30	Asymptomatic, mild	Saliva NP	PCR
Chen Y et al^16^	China	Retrospective cohort study	Hospitalized	42	Uncomplicated, mild, severe	NP Urine Stool	PCR
COVID-19 Investigation Team^11^	United States	Retrospective case series	Mixed – hospitalized and home isolation	12	Not defined	OP NP Serum Stool Urine	PCR Viral culture Whole genome sequencing
Danzetta ML et al^32^	Italy	Retrospective case series	Hospitalized and outpatient	14,200 tested; 605 positives	Not defined	NP OP	PCR
Di Tian LW et al^21^	China	Prospective case series	Hospitalized	75	Mild, moderate, severe, critical	Not specified	PCR
Fang Z et al^55^	China	Prospective case series	Hospitalized	32	ICU or non-ICU	NP Saliva Tears Blood Urine Stool	PCR
Fu S et al^24^	China	Retrospective case series	Hospitalized	50	Severe	NP Throat Rectal	PCR
Fu Y et al^27^	China	Retrospective case series	Hospitalized	410	Not defined	Throat	PCR
Gombar S et al^39^	United States	Retrospective case series	Not specified, but primarily outpatient	150	Not defined	NP	PCR
Han J et al^30^	China	Retrospective case series	Hospitalized	185	Mild, moderate, severe, critical	Respiratory	PCR
He X et al^64^	China	Prospective case series	Hospitalized	94	Moderate	Throat	PCR
Huang J et al^25^	China	Retrospective case series	Hospitalized	33	Moderate and severe	Throat Sputum Stool	PCR
Huang JT et al^29^	China	Retrospective case series	Hospitalized	308	General, severe, critically ill	Nasal Pharyngeal	PCR
Hung IF et al^41^	Hong Kong	Prospective case series	Hospitalized	9	Symptomatic and asymptomatic	NP Throat	PCR
Ikegami S et al^44^	United States	Prospective case series	Outpatient	272	Recovered	NP	PCR
Jiang X et al^74^	China	Case report	Home isolation	1	Asymptomatic	NP Anal	PCR
Kim ES et al^22^	Korea	Retrospective case series	Hospitalized	28	From 1 (no limit of activity) to 8 (death)	NP OP Sputum	PCR
Lan L et al^61^	China	Prospective case series	Hospitalized Home isolation	4	Asymptomatic Symptomatic	Throat	PCR
La Scola B et al^8^	France	Prospective case series	Hospitalized	155	Not defined	NP Sputum	PCR Viral culture
Le TQM et al^7^	Vietnam	Prospective case series	Hospitalized	12	1 asymptomatic, rest not specified	Throat	PCR Viral culture
Lee S et al^43^	Korea	Retrospective case series	Community treatment center	303	Symptomatic and asymptomatic	NP OP Sputum	PCR
Li N et al^48^	China	Retrospective case series	Hospitalized	36	Mild or severe	Respiratory Saliva	PCR
Li J et al^80^	China	Case report	Hospitalized	1	Mild/moderate	NP OP	PCR
Li W et al^37^	China	Retrospective case series	Hospitalized	18	Asymptomatic or mild	NP Nasal Sputum Throat Anal	PCR
Lin A et al^34^	China	Prospective case series	Hospitalized	137	Mild or severe	Not specified	PCR
Ling Y et al^47^	China	Prospective case series	Hospitalized	66	Not defined	OP Blood Urine Stool	PCR
Liu WD et al^12^	Taiwan	Case report	Hospitalized	1	Not defined	Sputum Throat Stool	PCR Viral culture
Liu Y et al^68^	China	Case report	Hospitalized	1	Severe	OP	PCR
Liu F et al^79^	China	Case report	Hospitalized	1	Moderate	NP	PCR
Lo IL et al^51^	China	Prospective case series	Hospitalized	10	Mild, moderate, or severe	NP Urine Stool	PCR
Long QX et al^38^	China	Point prevalence	Hospitalized	178	Asymptomatic, mild	NP	PCR
Min C et al^15^	Singapore	Retrospective case series and position statement	Not specified	766	Not specified	NP	PCR Viral culture
Miyamae Y et al^56^	Japan	Prospective case series	Hospitalized	23	Asymptomatic, mild	NP OP	PCR
Noh JY et al^35^	Korea	Retrospective case series	Residential treatment center	199	Asymptomatic or atypical symptoms	Not defined	PCR
Park SY et al^31^	Korea	Prospective case series	Hospitalized	6	Not defined	NP OP Sputum Saliva	PCR
Park SK et al^72^	Korea	Prospective case series	Outpatient	46	Asymptomatic and mild	Respiratory Stool	PCR
Pongpirul WA et al^19^	Thailand	Prospective case series	Hospitalized	11	Asymptomatic, mild, moderate	Upper respiratory	PCR
Qi L et al^54^	China	Retrospective case series	Hospitalized	147	Mild, moderate, or severe	Respiratory	PCR
Qian GQ et al^45^	China	Retrospective case series	Hospitalized	24	Not defined	Throat Rectal	PCR
Ridgway JP et al^42^	United States	Retrospective case series	Hospitalized and outpatient	555	Not defined	NP	PCR
Sakurai A et al^17^	Japan	Retrospective case series	Cruise ship	90	Asymptomatic	NP	PCR
Seah IYJ et al^76^	Singapore	Prospective case series	Hospitalized Discharged	17	Not defined	Tears NP	PCR
Sun J et al^66^	China	Prospective case series	Hospitalized	49	Mild or severe	NP Sputum Throat Stool	PCR
Talmy T et al^23^	Israel	Retrospective case series	Hospitalized	219	Mild	NP OP	PCR
Tan W et al^70^	China	Prospective case series	Hospitalized	67	Mild, moderate, severe	NP Sputum Blood Urine Stool	PCR
To KK et al^62^	Hong Kong	Prospective case series	Hospitalized	23	Mild or severe	OP Saliva	PCR
Van Kampen JJA et al^4^	Netherlands	Prospective case series	Hospitalized	129	Mechanically ventilated, ICU with oxygen therapy, ward with oxygen therapy, ward without oxygen therapy	NP Sputum	PCR Viral culture
Wang W et al^71^	China	Prospective case series	Hospitalized	205	Severe Nonsevere	Nasal Blood Sputum Stool Urine	PCR
Wang J et al^57^	China	Case report	Hospitalized	1	Mild	NP	PCR
Wang K et al^28^	China	Prospective case series	Hospitalized	68	CURB-65 scores 1–3	NP Sputum	PCR
Wang C et al^58^	China	Case report	Hospitalized	1	Mild	NP OP	PCR
Wang Y et al^86^	China	Prospective case series	Hospitalized	23	Mild and severe	NP Sputum Throat Fecal Urine Blood	PCR
Wölfel R et al^10^	Germany	Prospective case series	Hospitalized	9	Mild	Sputum NP Throat Stool	PCR Viral culture Whole-genome sequencing
Wu Y et al^20^	China	Prospective case series	Hospitalized	98	Not defined	Throat Stool	PCR
Xiao F et al^13^	China	Prospective case series	Hospitalized	28	Not defined	NP OP Stool	PCR Viral culture
Xiao AT et al^53^	China	Prospective case series	Hospitalized	56	Mild to moderate	NP Throat	PCR
Xu K et al^52^	China	Retrospective case series	Hospitalized	113	Mild, pneumonia, severe pneumonia, ARDS, septic shock	Sputum NP Throat BAL	PCR
Young BE et al^33^	Singapore	Prospective case series	Hospitalized	18	Not defined	NP Urine Stool Blood	PCR
Zhang L et al^69^	China	Case report	Hospitalized	1	Mild	Throat	PCR
Zhang J et al.^73^	China	Retrospective case series	Hospitalized	14	Not defined	OP Stool	PCR
Zhang WY et al^67^	China	Case report	Hospitalized	1	Mild	Pharyngeal	PCR
Zhang W et al^75^	China	Prospective case series	Hospitalized	39	Severe Nonsevere Clinical condition unknown	Oral Rectal	PCR
Zhang N et al^14^	China	Prospective case series	Hospitalized	23	Mild, moderate, critical	Upper respiratory Urine Stool Blood	PCR Viral Culture
Zhao F et al^36^	China	Retrospective case series	Hospitalized	401	Mild, moderate, severe	Respiratory Fecal	PCR
Zheng S et al^50^	China	Retrospective case series	Hospitalized	96	Mild, severe	Respiratory Blood Urine Stool	PCR
Zheng X et al^78^	China	Retrospective case series	Hospitalized	80	Common, severe	Throat Stool	PCR
Zhou B et al^49^	China	Prospective case series	Hospitalized	41	Severe	Throat	PCR
Zhou F et al^40^	China	Retrospective case series	Hospitalized	191	Mild, moderate, severe, critical	Respiratory	PCR
Zhou R et al^77^	China	Retrospective case series	Hospitalized	31	Asymptomatic	NP	PCR
Yao XH et al^59^	China	Case report	Hospitalized	1	Severe	NP Lung Liver Heart Intestine Skin	PCR Histopathology
Zou L et al^63^	China	Prospective case series	Hospitalized	18	Asymptomatic, mild-to-moderate, severe	NP	PCR

Note. NP, nasopharyngeal; OP, oropharyngeal; PCR, polymerase chain reaction; ET, endotracheal.

### Duration of Viral RNA Shedding

Overall, 77 reports included data on viral RNA shedding by PCR.^4-80^ Box 1 summarizes the key points of viral shedding duration. The duration of viral RNA shedding ranged from a minimum of 1 day^4,7,21,33,46^ to a maximum of 83 days.^48^ Intermittent PCR positivity did occur through day 92 from symptom onset in 1 case report—that patient had previously tested negative at day 72 followed by repeat positive PCR.^57^ In a study of 56 serially tested hospitalized patients with mild-to-moderate disease, 66.1% of NP and OP swabs were still positive at 3 weeks. Positivity rates then declined weekly and all PCR tests were negative by week 6.^15^ Based on the 28 studies that provided sufficient data (Appendix Table 1 online), the pooled median duration of RNA shedding from respiratory samples was 18.4 days (95% CI, 15.5–21.3). High heterogeneity was observed among these studies (I^2^ = 98.87%; *P* < .01).

**Box 1.** Brief Summary of Available Literature on SARS-CoV-2 Shedding

Duration of Viral Shedding in Respiratory Samples	Duration of Viral Shedding in Stool/Rectal Samples
The pooled median duration of viral RNA shedding in all severities of illness from respiratory isolates is 18.4 d (95% CI, 15.5–21.3). Intermittent RNA shedding up to 92 d after symptom onset has been observed. Viable virus has been isolated via culture from −6 to 20 d relative to symptom onset. Duration of RNA shedding exceeds the duration of viable virus shedding from 13 to 45 d.	Viral RNA shedding in stool has not been consistently observed. The pooled median duration of viral RNA shedding is 22.1 d (95% CI, 14.4–29.8). Viral RNA shedding up to 55 d after diagnosis has been observed. Viable virus has been isolated via culture of stool on day 19 of illness. Detection of stool RNA may lag behind detection of respiratory RNA by PCR.

We reviewed shedding data for patients with mild-to-moderate illness. Based on parametric regression modeling, Sun et al^66^ concluded that detection of viral RNA in throat swabs beyond 50 days post symptom onset in patients with mild illness would be a low probability event occurring beyond the 95^th^ percentile. Despite this calculation, there are case reports of patients with viral RNA shedding ≥45 days from symptom onset.^48,58,67-69,78,80^ Among all studies we reviewed, the longest duration of PCR positivity from a NP swab of a patient with mild illness was 92 days after symptom onset.^57^ The pooled median duration of viral RNA shedding from respiratory sources among patients with mild-to-moderate illness, based upon 10 studies that reported sufficient data (Appendix Table 1 online), was 17.2 days (95% CI, 14.0– 20.5). Again, there was high heterogeneity among these studies (I^2^ = 95.64%; *P* < .01).

There were multiple reports of patients with intermittently positive PCR results from respiratory specimens.^17,21,25,27,28,51,56-58,79,81^ Although not consistently defined, cessation of shedding was most often described as 2 consecutive negative PCR results ≥24–48 hours apart.^21,23,25,38,51,56,58,81^Tests were frequently done in anticipation of discharge from the hospital.^57,81^ One report estimated that 26%–49% of patients were positive again after a negative test, but in other studies re-positivity varied between 3% and 35%.^17,21,25,27,28,51,56,81^ Wang et al^57^ described a case report of a patient that was discharged 75 days after illness onset following 3 consecutive negative tests. The patient then tested positive on days 82 and 92, followed by negative PCR tests on days 101 and 105.^57^ Another case report described a woman with mild COVID-19 who intermittently tested positive by NP PCR swabs for 72 days from disease onset despite developing IgM and IgG antibodies on day 38.^58^

Wölfel et al^10^ observed that the pharyngeal rate of detection was highest in the first 5 days of symptom onset and then decreased.^10^ NP swabs may have a higher rate of detection than OP swabs, but they were only compared in 2 of the studies included in this review.^63,71^ Negative upper-tract specimens may not correlate with lower-tract specimens, though the significance of these findings is not well understood. In a postmortem analysis of a patient whose NP sample tested PCR negative, lung tissue was PCR positive and histology revealed coronavirus particles in bronchiolar epithelial cells.^59^

Some studies included data for presymptomatic or asymptomatic patients and observed that PCR positivity can occur as early as 5 days prior to symptom onset.^9,10,60,61^ Multiple case series reported that the viral load of asymptomatic patients are as high as those with symptoms.^9,10,62^ In one case series, the asymptomatic individual in a family cluster had similar viral RNA loads in nasal and throat swabs to those of symptomatic family members.^63^ The majority of the subjects in this case series converted to a negative PCR by day 18.^63^

In addition, 5 studies included saliva samples.^18,31,48,55,62^ In a series of 13 patients with mild disease, viral RNA load was highest in saliva in the first week of illness, but 3 of the patients still had detectable viral load in their saliva at day 20 of illness.^48^ In another series, PCR turned negative in the saliva of 13 mildly ill patients before nasal swab PCR: an average (±SD) of 13.33 ± 5.27 days and 15.67 ± 6.68 days, respectively.^55^ In the same study, the average duration of positive PCR in sputum was shorter in non-ICU patients than ICU patients, who were positive for an average (SD) of 16.5 ± 6.19 days.^55^

### Predictors of extended duration of viral RNA shedding in respiratory samples

The most frequently identified predictor of prolonged viral RNA shedding was disease severity. Patients with severe disease have been observed to shed RNA for longer and have higher viral RNA loads at symptom onset followed by a gradual decline in viral RNA 3 weeks after symptom onset.^29,32,50,53,64,65^ Based on 10 studies, the pooled median duration of viral RNA shedding from respiratory samples in patients with severe illness was 19.8 days (95% CI, 16.2–23.5) (Appendix Table 1 online). Again, significant high heterogeneity exists (I^2^ = 96.42%; *P* < .01). In one cohort of patients, the median duration (SD) of positive NP PCRs was 22.25 (±3.62) days in patients admitted to the ICU, compared to 15.67 (±6.68) days in non-ICU patients.^55^ Sun et al^66^ also observed prolonged duration of RNA shedding from NP swabs in those with severe illness compared to those with mild disease, with median durations of 33.5 days and 22.7 days, respectively.

Predictors of severe disease and duration of shedding ≥15 days in hospitalized patients included older age, hypertension, coronary artery disease, and diabetes mellitus.^17,27,50,52,53,62^ Gender was not consistently identified as a risk factor for severe disease or prolonged shedding but comparisons were limited by small sample sizes.^47,49,52,54,62^

### Viral RNA shedding in nonrespiratory samples

A subset of studies presented PCR data from both respiratory and fecal samples.^10-14,16,20,24,25,33,36,37,45,47,50,51,55,65,66,70-75,78^ Rectal/stool PCR pooled median duration of positivity based on 5 studies was 22.1 days (95% CI, 14.4–29.8; I^2^ = 95.86%; *P* < .01). Stool PCR positivity has been observed to lag behind both PCR positivity of pharyngeal specimens and symptom improvement and even may become positive after the OP PCR has become negative.^16^ RNA replication in the stool was observed ≥2 weeks after symptom onset.^10,20,50,51,73^ In one study, the number of PCR-positive stool samples increased between the first and third weeks of illness, with a median time to detection in the stool of 19–22 days.^50,70^ Based on the limited data available thus far, illness severity does not seem to impact stool RNA detection, as similar durations of RNA shedding in the stool have been observed in mild and severe illness.^16^ Park et al^72^ detected SARS-CoV-2 RNA in stool 50–55 days after initial diagnosis of asymptomatic or mild SARS-CoV-2 illness. In this study, people with higher viral loads were more likely to have viral RNA in the stool.^72^ However, stool shedding was not consistently observed, and some studies showed that virus was detectable in only 35%–59% of patients screened.^50,75^

Data for serum and blood are limited but are evolving. Among studies reporting serum or blood testing, viral RNA was detected in 30%–87.5% of patients with COVID-19, though a smaller study did not detect viral RNA in any of the 14 patients tested.^47,50,55,75,82^ The ability to detect RNA in blood and serum may be reflective of disease severity.^55,82^ Virus was detected by PCR for longer in blood samples of ICU patients [14.63 days (±5.88 SD)] compared to non-ICU patients [10.17 days (±6.13 SD)].^55^

### Correlation between viral culture and PCR

In total, 12 studies also included both PCR and viral culture information.^4-15^ Sequential viral cultures were not performed in all studies, which is a key limitation. Growth of SARS-CoV-2 on viral respiratory culture was reported ranging from 6 days before symptom onset through day 20 after symptom onset.^4,5,9,10^ A position statement published in Singapore reported that viable cultured virus was not isolated after day 11.^15^ Culture data suggest that the duration of shedding of viable virus may vary according to illness severity. In a study of patients with moderate-to-severe illness, Van Kampen et al^4^ found the median duration of shedding viable virus was 8 days (IQR, 5–11 days; range, 0–20 days) with the probability of detecting virus <5% after 15.2 days.^4^ In contrast, 4 studies of mildly ill patients did not find viable virus past day 8 or 9 of illness, but viral culture was not consistently reattempted.^5,9-11^ Liu et al^12^ described a patient with mild disease whose sputum viral culture was positive on day 18, but continued to have viral RNA detection until day 63, 45 days longer than detection of viable virus.

The correlation of SAR-CoV-2 viral loads and PCR cycle thresholds (Ct) values with isolation of viable virus is a topic of interest. The Ct value upper bound cutoff that determined a positive PCR was inconsistent among studies reporting this threshold, though most reported positive values at ≤35 or ≤40.^49-52,54,72,77^ Bullard et al^5^ compared PCR Ct value with culture positivity and found that the ability to isolate virus in culture was reduced when Ct value was ≥24. They reported that the odds ratio for infectivity decreased by 32% for every 1 point increase in the Ct value.^5^ La Scola et al^8^ report significant correlation between Ct value and culture positivity rates. Positive cultures occurred in all samples with Ct values 13–17 but culture positivity decreased to 12% at a Ct value of 33.^8^ Isolating virus in culture with positive PCR samples containing viral loads <10^6^ copies per milliliter is less likely to be successful.^4,10^

Limited data exist regarding SARS-CoV-2 cultures in nonrespiratory specimens. Viral culture was attempted in serum samples of PCR-positive patients without growth.^6^ Viral stool cultures have yielded mixed results. Wölfel et al^10^ performed viral culture of 13 stool samples from 4 different patients with mild disease on days 6–12 without growth, despite RNA detected in the stool through day 21. Viable virus was detected in the stool of a critically ill patient on day 19 with negative cultures beyond this despite a positive NP/OP PCR through day 28.^10^ Of 7 studies that processed urine samples, 2 reported detecting viable virus by culture.^11,47,50,51,55,62,71^ Also, 2 studies of patients with positive respiratory PCR samples attempted to culture virus from tears, but they yielded no growth.^55,76^

## Discussion

We summarized available data on duration of SARS-CoV-2 viral RNA shedding, isolation of viable virus, and the impact of infection severity on shedding duration. The pooled median duration of RNA shedding from respiratory samples of subjects was 18.4 days (95% CI, 15.54–21.3). In general, the highest viral loads occur within 1–2 weeks of illness onset, regardless of symptoms, with a subsequent gradual decline. However, several studies described PCR positivity beyond 2 weeks. Patients with more severe illness shed viral RNA for a longer period of time, with a pooled median duration of 19.8 days (95% CI, 16.2–23.5), compared to 17.2 days (95% CI, 14.0–20.5) for mild illness. Although these pooled medians should be interpreted with caution given the high heterogeneity of the studies and overlapping confidence intervals, viral culture data appear to support this conclusion. In reviewed studies, viable virus from respiratory cultures was not recovered past day 9 of illness for mildly ill patients but was cultured from severely ill patients through day 20.^4,5,9,10^

Interpreting positive PCR samples beyond 2–3 weeks of illness is complex. Potential explanations for these intermittently negative PCR tests include a viral load below the detection limit of the assay, specimen source, quality of specimen collection, timing of specimen collection or reinfection.^83,84^ Although viral culture positivity may also not correlate perfectly with transmissibility, the correlation between culture data and Ct thresholds may help predict infectiousness. Further data are needed to understand the correlation between transmission risk, culture positivity and Ct thresholds. The studies that examined viral culture were limited by small size, inclusion of patients with mostly mild illness, and lack of serial cultures on all patients. Isolation of viable virus in respiratory samples correlates with the timing of peak viral loads which occur within 1–2 weeks of illness onset. Only 1 study reported culturing viable virus from a respiratory sample beyond the second week of illness. Based on this information, it seems more likely that a positive PCR past 2–3 weeks of illness represents shedding of nonviable virus. Although the pooled median viral RNA shedding duration from patients with mild-to-moderate and severe disease do not differ greatly, reports of positive viral cultures through day 20 in severely ill patients support the potential for a prolonged infectious period for sicker patients. In addition, viable virus has been recovered from stool cultures, but further studies are needed to determine the implications for person-to-person spread.

Our review supports the US Centers for Disease Control and Prevention (CDC) interim guidance, which recommends maintaining transmission-based precautions for 10 days after symptom onset in asymptomatic or mildly ill patients and for 20 days in severely ill patients.^85^ The decision to extend the duration of transmission-based precautions is complicated given the potentially profound impact on patients and their families, hospital systems, and public health. Prolonged home isolation may lead to longer periods of unemployment, social separation, and feelings of isolation. In the hospital, the supply of personal protective equipment, staff allocation, availability of patient beds, and the health system budget are impacted by the duration of isolation for patients with COVID-19. That said, aggressive infection control measures are required in the setting of an outbreak to control the virus and to avoid overwhelming healthcare systems.

In calculating the pooled median duration of shedding, we identified a significantly high degree of heterogeneity between studies. In a standard meta-analysis, we would not report a pooled measure of association when heterogeneity was high. However, the pooled median is not intended to inform our knowledge of causality or effect size but, rather, to best inform the policy decisions that currently must be made on the very limited data available at this time in the SARS-CoV-2 pandemic. Factors contributing heterogeneity may include the variable timing of sample collection for PCR or viral culture, Ct threshold, sample types, SARS-CoV-2 genotype, and host factors such as pharmacotherapy, comorbidities, and disease severity. We noted broad variability in the definitions of disease severity applied. Although no formal definitions existed initially, the National Commission of China developed a classification scheme for mild, moderate, and severe illness that include specific clinical variables.^70^ The National Institutes of Health and World Health Organization have since developed similar severity scales also.^85,86^ Going forward, these definitions will facilitate the conduct of generalizable studies of viral dynamics.

This comprehensive review details the evidence available to date pertaining to SARS-CoV-2 viral dynamics. Although PCR positivity can be prolonged, culture data suggest that virus viability is typically shorter in duration. Continued reporting of viral shedding data via PCR and viral culture with improved standardization in methods and definitions, in coordination with transmission data, will facilitate evidence-based decision making for the infection control and public health measures necessary to control the pandemic.

## Supplementary material

For supplementary material accompanying this paper visit http://dx.doi.org/10.1017/ice.2020.1273. click here to view supplementary material
