# Supplementary material for: Understanding viral shedding of severe acute respiratory coronavirus virus 2 (SARS-CoV-2): Review of current literature
Source: Infect Control Hosp Epidemiol. 2020 Oct 20:1–10. doi: 10.1017/ice.2020.1273 (PMC7691645; doi:10.1017/ice.2020.1273)
Supplement: Supplementary file 1 [file S0899823X20012738sup001.docx]

**Appendix Table.** Studies contributing to calculations of pooled median duration of viral shedding of SARS-CoV-2

| Study | Disease Severity | Sample type | Days of Viral RNA Shedding | | | | | | | N | Inclusion in Pooled Median Analysis | | | |
| --- | --- | --- | --- | --- | --- | --- | --- | --- | --- | --- | --- | --- | --- | --- |
|  |  |  | **Mean** | **SD** | **Min** | **Q1** | **Median** | **Q3** | **Max** |  | **Respiratory Sample Data** | | | **Fecal/ Rectal Sample Data** |
|  |  |  |  |  |  |  |  |  |  |  | **All severity of illness** | **Severe Illness** | **Mild/Moderate Illness** |  |
| Chang M et al. | ND | Throat | -- | -- | -- | 4 | 5.5 | 8 | -- | 16 | ✓ |  |  |  |
| Chen Y et al. | All | Stool | -- | -- | -- | 7 | 11 | 13 | -- | 28 |  |  |  | ✓ |
|  | Mild | NP | -- | -- | -- | 6 | 8 | 10.5 | -- | 29 | ✓ |  | ✓ |  |
|  | Severe | NP | -- | -- | -- | 11 | 13 | 15 | -- | 11 | ✓ | ✓ |  |  |
| Danzetta ML et al. | All | OP/NP | -- | -- | 14 | -- | 30 | -- | 63 | 108 | ✓ |  |  |  |
| Di Tian LW et al. | Moderate | US | -- | -- | 1 | 9.5 | 25 | 42 | 63 | 45 | ✓ |  | ✓ |  |
|  | Severe | US | -- | -- | 2 | 9.21 | 14 | 21.25 | 62 | 20 | ✓ | ✓ |  |  |
| Fang Z et al. | Mild/Moderate | Nasal | 15.67 | 6.68 | -- | -- | -- | -- | -- | 24 | ✓ |  | ✓ |  |
|  | Severe | Nasal | 22.25 | 3.62 | -- | -- | -- | -- | -- | 8 | ✓ | ✓ |  |  |
| Fu Y et al. | All | Throat | -- | -- | 3 | 16 | 19 | 23 | 44 | 410 | ✓ |  |  |  |
| Han J et al. | Mild/moderate | NP/ sputum | -- | -- | 4 | -- | 16 | -- | 51 | 155 | ✓ |  | ✓ |  |
|  | Severe | NP/sputum | -- | -- | 4 | -- | 18 | -- | 51 | 30 | ✓ | ✓ |  |  |
| Huang J et al. | Moderate/severe | Stool | -- | -- | -- | 11.5 | 17 | 32 | -- | 33 |  |  |  | ✓ |
|  | Moderate/severe | Sputum | -- | -- | -- | 18.5 | 22 | 27.5 | -- | 33 | ✓ |  |  |  |
| Li N et al. | All | Saliva | -- | -- | -- | 47.75 | 53.5 | 60.5 | -- | 36 | ✓ |  |  |  |
| Lin A et al. | All | US | -- | -- | 4 | -- | 12 | -- | 45 | 137 | ✓ |  |  |  |
|  | Mild | US |  |  | 5 |  | 15 |  | 38 | 114 |  |  | ✓ |  |
|  | Severe/Critical | US | -- | -- | 4 | -- | 11 | -- | 45 | 23 |  | ✓ |  |  |
| Ling Y et al. | ND | OP | -- | -- | 2 | 6 | 9.5 | 11 | 22 | 66 | ✓ |  |  |  |
| Lo IL et al. | All | NP | 18.2 | 4.6 | -- | -- | -- | -- | -- | 10 | ✓ |  |  |  |
| Long QX et al. | Asymptomatic | NP | -- | -- | 6 | 15 | 19 | 26 | 45 | 37 | ✓ |  |  |  |
|  | Mild | NP | -- | -- | -- | 9 | 14 | 22 | -- | 37 | ✓ |  | ✓ |  |
| Miyamae Y et al. | Mild/asymptomatic | NP | -- | -- | -- | 6 | 19 | 37 | -- | 23 | ✓ |  |  |  |
| Pongpirul WA et al. | Mild/moderate | OP/NP | -- | -- | -- | 9 | 14 | 26 | -- | 11 | ✓ |  | ✓ |  |
| Qi L et al. | All | NP/Throat | -- | -- | 6 | 12 | 17 | 21 | 47 | 147 | ✓ |  |  |  |
| Qian GQ et al. | ND | Throat | -- | -- | 4 | 9 | 12 | 14 | 34 | 24 | ✓ |  |  |  |
| Sakaurai A et al. | Asymptomatic | NP | -- | -- | 3 | 6 | 9 | 11 | 21 | 90 | ✓ |  |  |  |
| Talmy T et al. | Mild | NP/OP | -- | -- | 4 | 15 | 21 | 27 | 45 | 219 | ✓ |  | ✓ |  |
| Tan W et al. | Mild/moderate | NP/Sputum | -- | -- | 3 | -- | 20 | -- | 33 | 38 | ✓ |  | ✓ |  |
|  | Severe | NP/Sputum | -- | -- | 7 | -- | 23 | -- | 38 | 29 | ✓ | ✓ |  |  |
| Wang K et al. | All | NP/sputum | -- | -- | -- | 16 | 21 | 31 | -- | 68 | ✓ |  |  |  |
| Wu Y et al. | ND | Feces | 27.9 | 10.7 | -- | -- | -- | -- | -- | 74 |  |  |  | ✓ |
| Xiao AT et al. | Mild/Moderate | NP/Throat | -- | -- | -- | 18 | 24 | 31 | -- | 56 | ✓ |  | ✓ |  |
| Xu K et al. | All | Sputum/NP/Throat/BAL | -- | -- | 13 | -- | 17 | -- | 32 | 113 | ✓ |  |  |  |
| Young BE et al. | All | NP | -- | -- | 1 | -- | 12 | -- | 24 | 18 | ✓ |  |  |  |
| Zhang N et al. | All | NP/throat | -- | -- | -- | 8 | 10 | 17 | -- | 11 | ✓ |  |  |  |
|  | All | Feces | -- | -- | -- | 15.5 | 22 | 23.5 | -- | 11 |  |  |  | ✓ |
| Zhao F et al. | All | Rectal | -- | -- | -- | 23 | 33 | 52 | -- | 80 |  |  |  | ✓ |
| Zheng X et al. | Severe | Throat | -- | -- | 14 | -- | 21 | -- | 30 | 74 | ✓ | ✓ |  |  |
| Zhou B et al. | Severe | Throat | -- | -- | 18 | 24 | 31 | 40 | 48 | 41 | ✓ | ✓ |  |  |
| Zhou F et al. | All | US | -- | -- | -- | 16 | 20 | 23 | -- | 191 | ✓ |  |  |  |
|  | Critical | US | -- | -- | -- | 22 | 24 | 30 | -- | 53 |  | ✓ |  |  |
|  | Severe | US | -- | -- | -- | 17 | 19 | 22 | -- | 66 |  | ✓ |  |  |

ND: Not defined

Min: Minimum

Q1: Quartile 1

Q3: Quartile 3

Max: Maximum

BAL: bronchoalveolar lavage

US: Unspecified respiratory source
